# Stakeholder perceptions of and attitudes towards problematic polypharmacy and prescribing cascades: a qualitative study

**DOI:** 10.1093/ageing/afae116

**Published:** 2024-06-09

**Authors:** Aisling A Jennings, Ann Sinéad Doherty, Barbara Clyne, Fiona Boland, Frank Moriarty, Tom Fahey, Larry Hally, Seán P Kennelly, Emma Wallace

**Affiliations:** Department of General Practice, University College Cork, Cork, Ireland; Department of General Practice, University College Cork, Cork, Ireland; Department of Public Health and Epidemiology, RCSI University of Medicine and Health Sciences, Dublin, Ireland; HRB Centre for Primary Care Research, Department of General Practice, RCSI University of Medicine and Health Sciences, Dublin, Ireland; Data Science Centre, School of Population Health, RCSI University of Medicine and Health Sciences, Dublin, Ireland; School of Pharmacy and Biomolecular Sciences, RCSI University of Medicine and Health Sciences, Dublin, Ireland; HRB Centre for Primary Care Research, Department of General Practice, RCSI University of Medicine and Health Sciences, Dublin, Ireland; Older People’s Council, Clare, Ireland; Department of Medical Gerontology, School of Medicine, Trinity College Dublin, Dublin, Ireland; Department of Age-related Healthcare, Tallaght University Hospital, Dublin, Ireland; Department of General Practice, University College Cork, Cork, Ireland

**Keywords:** polypharmacy, qualitative research, prescribing cascades, multimorbidity, medication reconciliation, older people

## Abstract

**Introduction:**

Problematic polypharmacy is the prescribing of five or more medications potentially inappropriately. Unintentional prescribing cascades represent an under-researched aspect of problematic polypharmacy and occur when an adverse drug reaction (ADR) is misinterpreted as a new symptom resulting in the initiation of a new medication. The aim of this study was to elicit key stakeholders’ perceptions of and attitudes towards problematic polypharmacy, with a focus on prescribing cascades.

**Methods:**

qualitative one-to-one semi-structured interviews were conducted with predefined key stakeholder groups. Inductive thematic analysis was employed.

**Results:**

Thirty-one stakeholders were interviewed: six patients, two carers, seven general practitioners, eight pharmacists, four hospital doctors, two professional organisation representatives and two policymakers. Three main themes were identified: *(i) ADRs and prescribing cascades—a necessary evil.* Healthcare professionals (HCPs) expressed concern that experiencing an ADR would negatively impact patients’ confidence in their doctor. However, patients viewed ADRs pragmatically as an unpredictable risk. *(ii) Balancing the risk/benefit tipping point*. The complexity of prescribing decisions in the context of polypharmacy made balancing this tipping point challenging. Consequently, HCPs avoided medication changes*. (iii) The minefield of medication reconciliation*. Stakeholders, including patients and carers, viewed medication reconciliation as a perilous activity due to systemic communication deficits.

**Conclusion:**

Stakeholders believed that at a certain depth of polypharmacy, the risk that a new symptom is being caused by an existing medication becomes incalculable. Therefore, in the absence of harm, medication changes were avoided. However, medication reconciliation post hospital discharge compelled prescribing decisions and was seen as a high-risk activity by stakeholders.

## Key Points

- All stakeholder groups considered adverse drug reactions and prescribing cascades to be unavoidable.
- The complexity of polypharmacy made balancing the medication risk/benefit tipping point very challenging.
- Prescribers avoided medication changes unless there was clear evidence of harm.
- Medication reconciliation post hospital discharge compelled prescribing decisions.
- Medication reconciliation was viewed as a perilous activity due to communication deficits and a lack of prescribing stewardship.

## Introduction

Polypharmacy, often referred to as being prescribed five or more medications daily [1], is common in older adults. The increasing prevalence of polypharmacy [2] is, in part, a consequence of increasing multimorbidity in an ageing population [3]. Polypharmacy can be a clinically appropriate response to multimorbidity. However, polypharmacy may lead to adverse outcomes, especially in older adults who are particularly vulnerable to the negative impact of polypharmacy due to age-related physiological changes. Polypharmacy becomes problematic for an older adult when the medicines prescribed are not clinically indicated or when they cause more harm than benefit [4]. Problematic polypharmacy can result in increased risk of hospitalisations [5], adverse drug reactions (ADRs) [6, 7] and unintentional prescribing cascades [8].

Prescribing cascades occur when a new medication is prescribed to treat or to prevent the symptoms of an ADR caused by another medication. Prescribing cascades can be intentional or unintentional. Intentional prescribing cascades would, for example, include prescribing a laxative to someone receiving opioid analgesia [9]. Unintentional prescribing cascades occur when an ADR occurs; the adverse effect is misinterpreted as a new symptom that results in the initiation of a new medication [10]. For example, a patient prescribed a diuretic because a prior prescription of a calcium channel blocker resulted in ankle oedema, which was misinterpreted as heart failure. Failure to recognise an ADR may result in an unintentional prescribing cascade, exposing the patient to continuing risk of an ADR from the culprit medication and additional risk from the newly prescribed medication [11]. Once recognised, the unintentional prescribing cascade can often be resolved relatively easily by deprescribing the offending drug [12]. The challenge is in recognising that the new symptom is being caused by a medication in the first place. In the context of polypharmacy in an older adult with multimorbidity, where there are several potential culprit medications and multiple underlying medical conditions, this process of identifying a prescribing cascade becomes even more complex.

Much research to date has focused on characterising potentially inappropriate prescribing using explicit prescribing indicators such as the Beers criteria [13] and the STOPP/START criteria [14]. However, much less is known about other elements of problematic polypharmacy such as prescribing cascades. Two recent systematic reviews have addressed this gap in both community-dwelling and in-patient populations. These reviews reported multiple examples of prescribing cascades described in the literature. Examples include the prescription of loop diuretics for pedal oedema related to calcium channel blocker prescription and levothyroxine prescribed for amiodarone-induced hypothyroidism [15, 16]. An explicit set of prescribing cascades *ThinkCascades* has recently been published and includes nine clinically relevant prescribing cascades agreed through international expert consensus [17]. Yet, the need to integrate prescribing cascades with other inappropriate prescribing criteria is recognised [8].

To develop effective interventions in this area, the challenges of identifying and avoiding prescribing cascades need to be fully explored and understood. Previous research has explored polypharmacy from the perspective of patients and general practitioners (GPs) [18, 19]. More recently, research has specifically explored GPs’ and patients’ experiences of prescribing cascades in the context of polypharmacy [20, 21]. However, no study has taken a fully integrated approach and explored problematic polypharmacy and unintentional prescribing cascades from the perspective of all the relevant stakeholders. A stakeholder analysis is necessary to provide a comprehensive understanding of the complexity of the problem, which will help to inform effective interventions in this area [22].

The aim of this study was to conduct a stakeholder analysis (patients with polypharmacy; carers; GPs; hospital doctors; community pharmacists; professional organisation representatives and policymakers) to elicit perceptions of and attitudes towards problematic polypharmacy, with a focus on prescribing cascades.

## Methods

One-to-one semi-structured interviews were undertaken with a broad range of stakeholders in Ireland. The method of analysis was inductive thematic analysis as outlined by Braun and Clarke [23]. Results are reported following the consolidated criteria for reporting qualitative research (COREQ) guidelines (Appendix 1). Ethical approval was obtained from the Irish College of General Practitioners Research Ethics Committee (ICGP_REC_21_0011) and the Royal College of Surgeons Ireland (RCSI) University of Medicine and Health Science Ethics Committee (Record ID: 212553532).

### Sampling and recruitment

Participant recruitment was conducted using a gatekeeper approach, employing convenience and snowball sampling. For GP recruitment, the study was advertised to members of the Irish College of General Practitioners, the national professional organisation for GPs in Ireland, during a continuing professional development (CPD) webinar (~1,000 attendees). Those who were interested in study participation, as either a GP participant or to act as a gatekeeper to support patient and carer recruitment, were invited to contact a member of the study team (AD) to obtain further information on the study. Patients and carers were recruited using GPs as gatekeepers. The GPs who acted as gatekeepers were recruited from three possible sources. Firstly, GPs who attended the CPD webinar and contacted the research team expressing an interest in supporting patient/carer recruitment although they did not act as interview participants themselves. Secondly, GPs who participated in the interviews and subsequently agreed to act as gatekeepers for patient and carer recruitment. Thirdly, GPs who were members of the University (RCSI) GP Tutor Network were contacted via email and invited to act as gatekeepers for patient and carer recruitment. Using a gatekeeper approach has been shown to be helpful in recruiting participants to qualitative research, particularly in clinical settings [24]. The GPs contacted suitable patients and carers based on the inclusions and exclusion criteria outlined in Table 1. The GPs provided interested patients and carers with a study pack that included a patient information leaflet, an invitation letter and a consent form. The patients/carers then returned the consent form to the research team via post if they were interested in participating. Community pharmacists were recruited using a gatekeeper who advertised the study via a CPD network of community pharmacists (Irish Institute of Pharmacy Peer Support Pharmacist Network). Hospital doctors, including consultant geriatricians and non-consultant hospital doctors, were recruited via gatekeepers affiliated with the RCSI University of Medicine and Health Sciences in Dublin, Ireland. Professional organisation representatives and policymakers were recruited to understand the sociocultural influences on prescribing and medication management at a more national level. The representatives could speak to education and training and broader policy influences that shape medicines management. Policymakers and representatives of professional organisations were recruited via contacts available within the study team. Study team members asked these primary contacts to circulate an email amongst eligible staff members/colleagues on behalf of the study team to ascertain interest in study participation. Further information on interview participants is available in Table 2. Study materials were distributed by email (healthcare professional stakeholders) and by post (patients and carers, distributed by their GP gatekeeper). Once informed consent was obtained, direct contact was made with participants to arrange an interview time.

**Table 1:** Inclusion and exclusion criteria for patients and carers

Inclusion criteria for patients	Patients who were aged ≥65 years and who were prescribed at least 10 regular medications and who were able to give informed consent
Exclusion criteria for patients	Patients (<65 years) Patients with cognitive impairment to the level that would impede their ability to give informed consent Patients who are recently bereaved (<1 month) Patients with active psychotic illness Patients in nursing homes or residential care
Inclusion criteria for carers	Carers who look after an older person (>65 years) prescribed >10 regular medications
Exclusion criteria for carers	Carers who do not support an older adult with polypharmacy

**Table 2:** Information on interviews and interview participants

Stakeholder group	Number of participants	Demographics	Average interview length
**Pharmacists**	8	Working in an urban area (*n* = 3) Working in a mixed rural/urban area (*n* = 3) Working in rural area (*n* = 2)	65 min
**General practitioners**	7	Urban practice affluent area (*n* = 1) Urban practice mixed deprivation and affluence (*n* = 2) Urban practice area of significant deprivation (*n* = 2) Rural practice mixed deprivation and affluence (*n* = 2)	62 min
**Patients**	6	Average age 76 (range 68–86 years) Average number of regular medications was 16 (range 12–23 regular medications)	53 min
**Carers**	2	Female, aged 42, live in carer for family member living with advanced dementia who is on 13 medications Female, aged 31, live in carer for family member living with dementia who is on 15 medications	67 min
**Hospital interns**	3	All 3 participants had completed 12 months of Hospital Internships and were imminently commencing hospital posts as Senior House Officers One participant had worked as a medical intern in gastroenterology and rheumatology One participant had worked in orthopaedics and had experience with orthogeriatrics as a result One participant had worked as a medical intern with endocrinology and general medicine teams	68 min
**Consultant geriatrician**	1	Working in a University Teaching Hospital	62 min
**Policymakers**	2	Both participants had a background in clinical pharmacy in the hospital setting. One of the policymakers also had some experience in the community setting	91 min
**Professional organisation representatives**	2	One participant was an experienced practising GP who was working in a leadership role within the Irish College of General Practitioners One participant was an experienced Community Pharmacist and was working in a leadership role within a Professional Organisation for Pharmacists in Ireland	60 min

### Data collection

**Figure 1:**
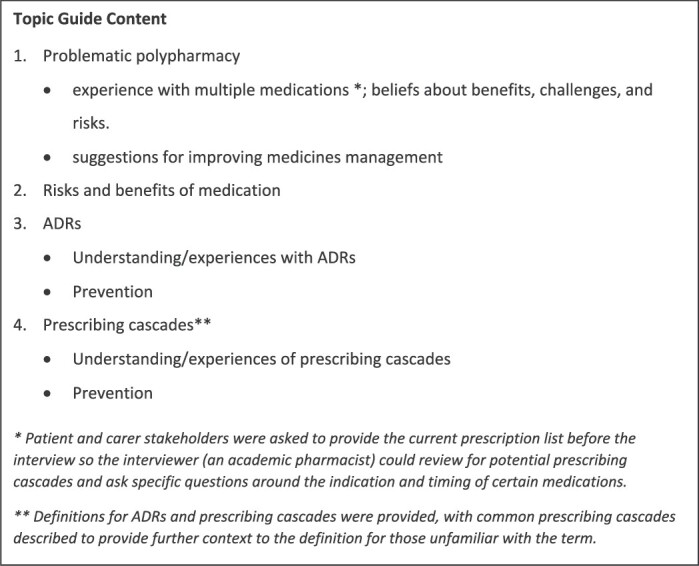
Topic guide content.

The topic guide was developed in consultation with the wider multidisciplinary research team with input from a PPI (Patient and Public Involvement in research) advisor. The topic guide explored four key areas as outlined in Figure 1. The topic guide is available as Appendix 2.

Interviews were conducted by an academic pharmacist (AD) using either Microsoft Teams or telephone from April to October 2021. The principles of information power were used to determine when a sufficient number of interviews had been conducted within each stakeholder group [25]. Interviews were transcribed verbatim via a professional transcription service. Transcripts were made available to participants for review prior to data analysis, where requested. Participants received a voucher for €100 as an acknowledgement of the time taken to participate in the interview. Participants were pseudonymised according to their primary stakeholder category and in order of study recruitment (e.g. GP1–GP7, Pharm1–Pharm8 etc.). De-identified transcripts were verified for accuracy against audio recordings (AD). NVivo 12 was used to manage the data.

### Reflexivity

The project team included GPs, pharmacists and a geriatrician. However, there were also healthcare researchers who had no clinical background and a PPI contributor on the research team. Decisions about recruitment approaches, selection of potential professional gatekeepers and approach to analysis were conducted at the project team level.

An extensive familiarisation process was conducted by two members of the research team (AJ, AD) who read and re-read all the transcripts. AJ is a GP and qualitative researcher. AD is a pharmacist and a postdoctoral researcher in problematic polypharmacy. AJ open coded all the transcripts. AD conducted the interviews and coded a purposively selected subset of interviews that included two interviews from each stakeholder group. Reflective notes were taken during data analysis to facilitate data interpretation and transparency in coding and theme generation. We recognise that qualitative research is not conducted in an epistemological vacuum; it is, therefore, inevitable that the researcher’s own professional backgrounds influenced how the data were interpreted [26]. These potential influences were acknowledged in regular meetings throughout the data analysis process. During these meetings, the researchers (AJ and AD) frequently considered their own professional biases and how these biases could shape the interpretation of certain interviews. For example, it was recognised that an interview that was critical of the researcher’s own profession could have impacted on how that interview was coded and warranted further analysis by the second researcher. Similarly, interviews that were not aligned with the researcher’s experience of their own professional identity were subject to further analysis. This process helped to address potential professional biases by introducing the perspective of another researcher from a different profession. Prior to finalising the codes, four members of the multidisciplinary team met to discuss the data findings and code development (AJ, AD, EW, BC). EW is an experienced academic GP. BC is an experienced qualitative researcher with an interest in medicines-related research in primary care and also provided the perspective of a non-clinician. Raw data, coding structure documents, summary memos, meeting minutes and theme reports were filed by date to comprise an audit trail of the analysis process. The final themes were discussed in detail with our PPI contributor (LH).

Four participants had prior contact with AD through various professional activities, e.g. CPD training, shared employer or interview recruitment panels. These four participants were aware that AD was the primary point of contact within the study team prior to consenting to participate in the study. Participants were aware that AD was an academic researcher and pharmacist. Other team members involved in data analysis (AJ, EW, BC) had no contact with study participants.

## Results

Thirty-one interviews were conducted as outlined in Table 1. Interviews were, on average, 64 min in duration (range 40 to 108 min).

Three main themes were identified.

(1) ADRs and prescribing cascades; a necessary evil
(2) Balancing the risk/benefit tipping point
(3) The minefield of medication reconciliation

**Figure 2:**
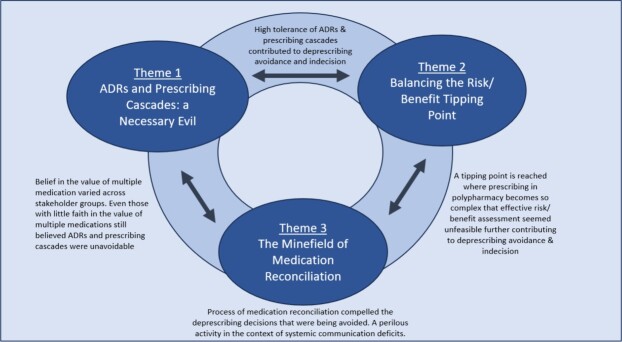
Overview of the three themes.

The three themes and how they integrate are outlined in Figure 2.

### Theme 1: ADRs and prescribing cascades a ‘necessary evil’

The stakeholder groups had different beliefs in the benefit of medication in the context of polypharmacy. From the perspective of the healthcare professionals, pharmacists tended to believe strongly in the importance of medication adherence. Some of the pharmacists interviewed used quite definitive language when discussing the value of medications.

*If they are prescribed something they should start on the treatment anyway. No matter how many medications—you have to take them. (Pharm1)*

Within this context of strong faith in the benefit of medication, ADRs and prescribing cascades were seen as unavoidable by pharmacists.

*a necessary evil you know like because the first medicine is essential, you know and there is no getting around it. (Pharm7)*

The GPs and consultant geriatrician interviewed did not share the pharmacists’ faith in the benefit of medication in the context of polypharmacy, with the participating consultant geriatrician stating that there was ‘*no benefit to taking multiple mediations’.* The GPs’ attitude to polypharmacy tended to be more nuanced with an appreciation of the challenges inherent to managing multimorbidity. However, like the pharmacists, GPs perceived ADRs and prescribing cascades as unavoidable consequences of polypharmacy. Although healthcare professionals were willing to tolerate ADRs and unintentional prescribing cascades they expressed concern that experiencing these would negatively impact the patient’s trust in medication and confidence in their doctor.

*It kills the confidence in the person who prescribed it. So, they might lose trust in their doctor, they might lose trust in their other medicines and start getting anxiety around their tablets. (Consultant Geriatrician)*

However, contrary to the concerns of the HCPs, the patients and carers interviewed had a very pragmatic approach to both ADRs and unintentional prescribing cascades. ADRs were viewed by patients and carers as an unpredictable risk that did not impact on either their trust in medications or in their doctors.

*You can’t really know. Because everybody’s different. So, it’s very hard to, if I take a tablet and you take a tablet. We react different, so it’s very hard to pre-empt it. (Patient1)*

Although ADRs were viewed as undesirable events that *‘throw a spanner in the works of everyday life’* (Carer2), patients and carers felt it was a risk that needed to be tolerated to reap the potential benefit of the medication. This high level of tolerance occurred in the context of patients’ and carers’ trust in their doctor’s prescribing. The patients interviewed believed that the medications that their doctors prescribed had a purpose and kept them alive. The interviews with patients had a more deferential tone and spoke of their faith in both their medications and the doctors prescribing them. They trusted that their doctor had their best intentions at heart when they made prescribing decisions.

*I would trust my doctor a million percent if there was such a thing. (Patient3)*

Prescribing cascades were viewed as a more nebulous concept for patients and carers than ADRs. The concept of an intentional prescribing cascade, when explained with examples, was understood by patients and carers, and viewed as necessary and reasonable. However, in the context of their absolute trust in their doctor’s prescribing decisions, some patients struggled to understand the concept of an unintentional prescribing cascade.

*Well, the reason to prescribe the cascade is to help you. Because you need to take the other tablet, unfortunately the side effect is your feet, or you know something else. I don’t think doctors prescribe you tablets unless you need them. (Patient1)*

### Theme 2: balancing the risk/benefit tipping point

Prescribing decisions in the context of polypharmacy was characterised as a careful balancing act that required constant trade-offs. Participants described a mindful approach to prescribing that required weighing up the risk: benefit of each new medication commenced. However, the prescribing scales in polypharmacy was not easy to balance.

*Well, everything you prescribe you add to the risk you know. The risk is, it’s not even cumulative, sometimes it’s you know multiplicative; you’re multiplying the risk. . . . are you tilting from benefit to risk*. *(Pharm4)*

Several GPs experienced prescribing in patients with polypharmacy as an ethical dilemma.

*You almost have this battle in your head, do you do more harm than good by stopping stuff or do you carry on. Because the patient may not have come to any harm up to that point, you know. (GP1)*

Initiating a new medication or deprescribing an existing medication risked disrupting the *‘very unsteady equilibrium’* (GP7) and tipping the scale unfavourably into risk. When a patient was experiencing intolerable side effects, the decision to deprescribe was straightforward. The challenge for prescribers occurred where there was a potential risk and a potential benefit to stopping the medication. This created a ‘deprescribing inertia’ that potentially contributed to the creation of unintentional prescribing cascades. Participants provided many examples of situations where patients were prescribed multiple conflicting medications. Sometimes these prescribing cascades were presented as unintentional errors but other times they were presented as prescriber indecision, a reluctance on the part of the prescriber to choose what condition to treat and what condition to not treat.

*You know, when you see people on both—there’s a drug that raises your blood pressure and then a tablet, a little smidgen of a blood pressure lowering tablet. It’s completely non-sensical but loads of people do it. Because they just don’t want to deal with the decision of not treating one of them. . . . (Consultant Geriatrician)*

Making prescribing decisions that had risk benefit trade-offs required a level of understanding, knowledge and time. All healthcare professional groups referenced how challenging it is to correctly identify that a new symptom is being caused by an existing medication.

*I suppose when you introduce a cocktail of medicines, if someone isn’t feeling well in themselves for whatever reason whether it's nausea or just lethargy it is very, very difficult to pinpoint whether it's a disease state or if it's a particular medicine causing a particular side effect you know. (Pharm7)*

Furthermore, one of the professional representatives described how it is potentially challenging for doctors to consider an unintentional prescribing cascade as the cause of the patient’s symptoms because it involves the doctor unravelling a previously solved problem.

*If they have to start going back and second-guessing decisions that they have in their mind ticked off as already having made. . . . if every time somebody is coming with something new, we have to go back to the start. You know I can see why that doesn’t appeal to people. (Prof Rep1).*

Overall, the key barrier to identifying prescribing cascades appeared to be the sheer complexity of polypharmacy. At a certain level of polypharmacy, the risk that a new symptom is being caused by an existing medication becomes incalculable. A tipping point is reached where prescribing in the context of polypharmacy becomes so complex that effective risk assessment seemed unfeasible. The complexity inevitably tipped the scale towards risk.

*It just becomes so complex, and it means that all of the hazards are, it's just all hidden. It's so difficult for anybody to kind of think clearly once you are into that level of polypharmacy. (Policymaker1)*

### Theme 3: the minefield of medication reconciliation

Managing the medications of an older adult with polypharmacy post hospital discharge was an emotive issue for GPs, pharmacists, patients and carers. GPs viewed the reconciliation of medications in patients with polypharmacy post hospital discharge as a perilous activity; *‘an absolute lethal minefield’ (GP2)*. The point of medication reconciliation compelled prescribing decisions. There was little room for avoidance or indecision. Yet, the activity was perceived to be fraught with danger. Medication changes that occurred within secondary care were primarily communicated to the GP via discharge letters. GPs were reliant on these discharge letters for information on medication changes since the hospital prescription generally went directly to the pharmacy. However, GPs reported that the discharge letters were frequently delayed and information on medication changes was often incomplete. The most junior member of the hospital in-patient team was recognised as being responsible for completing the discharge letters and prescriptions. Both the Hospital Interns and the GPs interviewed described significant variation in the completion of discharge letters. The Hospital Interns outlined how the clinical information included in the discharge letter was dependent on the diligence and the capacity of the Intern. Discharge letters often had a designated box to document medication changes; however, these boxes were not routinely completed.

*As long as you remember to fill in that section at the bottom. We don’t always. It does get missed, because you have to take time to look at what they were on in the community and then what are they on now and do changes. So, if you remember to fill it in. . . (Hospital Intern1)*

This delay and lack of adequate information was a specific challenge in the context of older adults with polypharmacy. These hospital admissions tended to more complex and resulted in more medication changes. The patients were at high risk for ADRs and often were unable to accurately communicate what medications had been changed. In the context of delayed, and sometimes incomplete, information post hospital discharge, the GPs relied upon the community pharmacist for information on medication changes. In polypharmacy, where there are often multiple prescribers, the pharmacist was seen as the infallible backstop that mopped up what was lost in translation. From the perspective of the pharmacists, medication reconciliation in the context of polypharmacy was seen as a challenging and high-risk endeavour. Pharmacists outlined the many potential sources of error that could occur in medicine reconciliation in a person with polypharmacy. Like some of the GPs, pharmacists used the term *‘minefield’* when discussing this task. The process of accurately identifying the correct list of medications for a patient with polypharmacy required the pharmacists to pro-actively pursue information rather than simply passively receiving it.

*It's a complete mess. It’s like you have to become a detective and work your way back because we’ll never find out like, it's a nightmare. (Pharm6)*

The pharmacists did not feel as infallible as the other healthcare professionals perceived them to be. Pharmacists felt frustrated that had no access to the discharge letter or to relevant clinical information.

*I find that sometimes your hands are tied a lot. Because you don’t have a lot of information. You’re sort of relying on people and it feels like you’re taking a risk when you make these decisions sometimes. (Pharm3)*

The potential for medication error during the process of medication reconciliation was recognised by healthcare professionals and carers. As was the risk of the patient *‘falling through the cracks’ (Pharm6, Prof Rep1).* Carers described a hypervigilant state, constantly checking that the prescription was correct and monitoring for medication side effects. The carers felt that the burden of identifying unintentional prescribing errors fell to them because healthcare professionals were busy.

*. . . you have to be quite on the ball like because things can get lost in translation. . . You know they didn’t get the updated prescription, so that they’re giving you the wrong doses. . . So, you do have to keep an eye on it. . . it could slip through the net. (Carer1)*

The patient and/or carer was simultaneously central to and excluded from this ‘pharmacy-GP–secondary care’ communication triad. They were required to compensate for the systemic communication deficits by relaying relevant information to the different members of the communication triad. However, they did not feel adequately informed to act as this conduit for prescribing information.

*They [hospital doctors] just give me a prescription and say bring that to your GP. I think, depending on the GP to give you the talk about what the medicine is and all that. But like that’s not facing facts, the fact of life in a modern GP. . . . doctors don’t have the time to sit down and talk to me about my latest medicine. (Patient2)*

**Figure 3:**
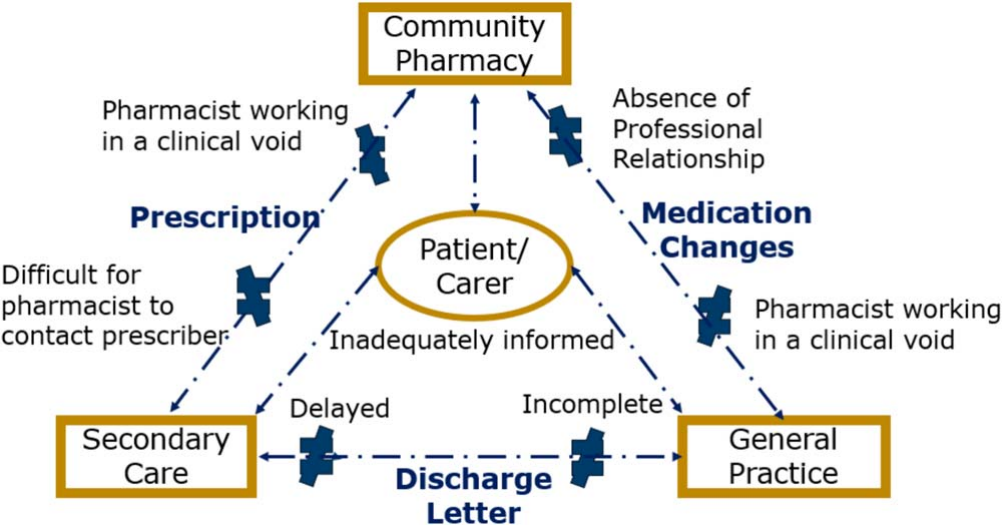
Barriers to an effective medication reconciliation process.

No stakeholder group felt they had access to adequate, timely information to facilitate the safe reconciliation of medication in a patient with polypharmacy (see Figure 3). They felt this was a risk that they had to carry in the context of systemic failures at points of transitions of care. Each stakeholder group referenced the problems caused by the lack of ownership and oversight over the entirety of the prescribing, but they were uncertain about which professional group should provide this prescribing stewardship. GPs and pharmacists both felt that they were responsible for untangling the web of prescribing created by the different prescribers involved in the care of patients with polypharmacy but questioned if this should be their responsibility.

*I don’t know who but there probably should be one sort of gatekeeper, you know. So, I don’t know who that gatekeeper is. Is it meant to be the GP or is it the pharmacist? But if there was one sort of gatekeeper that was doing an overall view of their medicine. (Pharm2)*

A clinical pharmacist working in the GP practice providing expertise in medication reviews was suggested by many participants as the most appropriate professional to provide the stewardship necessary for patients with polypharmacy.

## Discussion

In this stakeholder analysis of patients, carers, GPs, community pharmacists, hospital-based doctors and policymakers, three main themes were identified. First, ADRs and prescribing cascades were considered unavoidable for older patients living with multimorbidity taking multiple long-term medicines. Second, for clinicians, balancing the risk-to-benefit tipping point for individual patients is very challenging and requires integration of evidence, clinical experience and patient preferences. Third, challenges in medicines reconciliation following hospital discharge represent a time of increased risk of medication-related problems for patients, prescribers and community pharmacists.

### Comparison with existing literature

Stakeholders viewed ADRs as inevitable and unavoidable in the context of complex polypharmacy in an older adult. GPs expressed concern that if a patient experienced an ADR, this would negatively impact the patient’s trust in medication and confidence in their doctor. However, patients and carers had a very pragmatic approach to ADRs and unintentional prescribing cascades. They saw these as unpredictable risks that did not impact on either their trust in medications or in their doctors. The centrality of a trusting relationship with healthcare professionals was also highlighted by patients in a previous UK qualitative study [27] and in a qualitative synthesis of patients perspectives on reducing polypharmacy [18]. Overall, there has been relatively little research examining patients’ perceptions of and experiences of ADRs. A systematic review (*n* = 33 studies) examining the broader issue of patient-reported adverse events highlighted medication errors, communication failures and care coordination issues [28]. Patients reported experiencing distress after an adverse event and not receiving adequate information from healthcare professionals. However, results were limited by small study sample sizes and varying definitions of what constitutes an adverse event [28].

Different stakeholders held different views on the necessity of medicines in the context of managing multimorbidity. Pharmacists and patients viewed medicines as essential, whereas GPs and the consultant geriatrician interviewed described the inherent challenges of managing polypharmacy in the context of multimorbidity and the precarious risk-to-benefit balance involved. For GPs, unless there was clear evidence of harm, medication changes were often avoided, creating a deprescribing inertia. This finding is in line with previous qualitative research where GPs integrated information from multiple sources including the patient, specialists and evidence-based medicine in their management of patients with multimorbidity and polypharmacy [29]. Difficulties arose when recommendations or preferences conflicted, to which GPs responded by adopting management approaches that they deemed satisfactory on a patient-by-patient basis. GPs preferred to ‘maintain the status quo’ for stable multimorbid patients rather than rationalise medications, even with significant polypharmacy [29].

Prescribing cascades were presented as occurring either because of a failure to identify a cascade or as a result of prescriber indecision. Participants believed that preventing avoidable prescribing cascades required time and necessitated a thorough medication review, which required knowledge of potential prescribing cascades. Similarly, a recent qualitative study with healthcare professionals and patients on prescribing cascades found that an ability to identify a prescribing cascade, as distinct from a new symptom, was key to the prevention of prescribing cascades [21]. Research to date has sought to identify lists of potential prescribing cascades [15–17]. More recent research has systematically assessed the evidence supporting these potential prescribing cascades [30]. Findings from these studies need to be translated into educational initiatives to raise awareness of potential prescribing cascades. In our study, participants described how, even if a prescribing cascade was identified, the clinician was then required to weigh up whether the benefit of continuing a prescribing cascade outweighed the risk of potential harm. This process of risk–benefit assessment was presented as being particularly difficult in the context of an older adult with multimorbidity and polypharmacy. For these patients, the risk/benefit tipping point was delicately balanced and easily disrupted. A previous qualitative synthesis that explored GPs’ perspectives on reducing polypharmacy found that weighing the risk of medication side effects against the risk of discontinuing them, and striking the optimum balance, was very challenging for GPs [18]. A previous commentary has described how cursory-risk benefit assessments and inadequate documentation can contribute to problematic prescribing cascades [31]. A recent qualitative study on patients’ and providers’ experiences of prescribing cascades identified that the same prescribing cascade could be interpreted as being problematic or appropriate, depending on the complexity inherent to the individual prescribing cascade [20]. In our study, we identified that once a certain depth of polypharmacy was reached, HCPs believed that using a risk/benefit assessment to determine whether a prescribing cascade was appropriate or problematic was unfeasible due to the level of complexity it involved.

Medication reconciliation after hospital discharge compelled prescribing decisions and was viewed as a perilous activity. Communication failures and a lack of prescribing stewardship inherent to the medication reconciliation process across secondary/primary care transitions further added to the burden of risk that stakeholders felt. Challenges encountered included delays in GPs receiving discharge summaries/handovers from hospital, inadequate or absent information relating to medications on discharge summaries and a lack of a shared electronic health record. Both the Hospital Interns and GPs interviewed described significant variation in the completion of discharge letters, including documentation of medication changes. Transitions of care are recognised as a time of increased risk for patients and has been identified as a central theme in WHO’s Medication Without Harm agenda [32]. A UK study that examined safety issues related to patient transitions from hospital to primary care using national incident reporting data (*n* = 598 reports) identified areas where deficits led to patient harm [33]. These included errors in discharge communication, errors in referrals to community care teams and medication errors [33]. A recent UK Delphi consensus study identified key threats to safe patients transitions from secondary to primary care [34]. ‘*Poor quality of handover instructions from secondary to primary care teams’* achieved the highest rating and 100% consensus that it was a *‘very important’* threat. Older individuals and patients with complex medical problems taking >5 long-term medications were considered most vulnerable [34].

Due to communication failures across care transitions, GPs in this stakeholder analysis relied on community pharmacist and patients/carers for information regarding any medication changes that occurred while an in-patient. From the perspective of pharmacists, medication reconciliation in the context of polypharmacy was seen as a challenging and high-risk endeavour. GPs and community pharmacists were not clear on who held or should hold overall responsibility for this activity. Stakeholders agreed on the central role of the community pharmacist in supporting medication safety across care transitions. This finding is in line with the results of a systematic review that examined the effectiveness of interventions (*n* = 4 studies) that included community pharmacists in secondary to primary care transitions and reported improvements in the identification and rectification of medication errors [35].

### Strengths and limitations

The extensive sampling process provided in-depth access to multiple perspectives on problematic polypharmacy. The interviews were of a substantial duration and depth. This provided rich insights to our research question. Reflexivity was embedded in the data collection and analysis process, thus increasing the transparency and trustworthiness of the research. The data analysis team had diverse professional backgrounds from General Practice (AJ and EW), Pharmacy (AD) and Population Health and Health Services Research (BC). This use of analyst triangulation helped to decrease professional biases in the analysis process and increased the confirmability of the findings. The structured approach to data analysis with a detailed audit trail increases the dependability of the research findings.

The sampling strategy, although robust, had some potential limitations. The patients and carers recruited to this study were identified via a GP gatekeeper. The patients and carers interviewed all expressed high levels of trust in the medical profession, particularly their GPs. It is possible that when identifying patients to recruit to the study, the GPs identified patients and carers with whom they had a strong relationship of trust. However, previous research with older adults has likewise found that this patient cohort has a high level of trust in their GPs [36, 37]. Although extensive recruitment strategies were applied, only one Consultant Geriatrician was interviewed. The recruitment process was conducted during the height of the COVID pandemic and at a time where the Irish health service was targeted by a criminal cyber-attack [38]. This made recruitment of Consultant Geriatricians challenging. Although the one interview conducted with the Consultant Geriatrician exceeded 1 hour in duration and provided rich insights, further exploration of the perspective of this key stakeholder group would be desirable.

### Implications for research and practice

In this stakeholder analysis, the key challenge for HCPs when managing unintentional prescribing cascades was detecting an unintentional prescribing cascade in the first instance. This challenge was made particularly difficult given the ambiguous presentation of ADRs in an older adult with polypharmacy. There is a role for educational interventions to improve recognition of commonly encountered adverse drug reactions. However, education alone is not sufficient if HCPs are not resourced to implement the best practice that the education recommends. Time has been identified as a significant barrier to HCPs engaging with continuous professional development [39, 40]. The participating HCPs suggested a structured medication review would help to identify unintentional prescribing cascades but recognised that there is no dedicated time for GPs to conduct these reviews. The concept of the medication reviews being conducted by a GP-based pharmacist rather than by the GP was suggested by several participants. A recent qualitative study explored the implementation of a pharmacist-led structured medication review in General Practice in the UK [41]. This structured medication review aimed to alleviate workload pressures on GPs. However, the study found that the medication reviews were generally not conducted as intended and were often delivered in an ad hoc, reactive manner. Understanding and addressing the implementation factors that prevent the delivery of such interventions is important for future research in this area to prevent repeating similar studies in different jurisdiction with the same sub-optimal outcomes.

The importance of patient education and empowerment was identified. In this stakeholder analysis, patients were both central to and excluded from the communication pathways that were crucial to safe medication reconciliation. The importance of educating patients on their medications and empowering them to identify ADRs and unintentional prescribing cascades was highlighted by multiple stakeholder groups. A recent systematic review examined how best to support older adults during care transitions to reduce medication-related problems (*n* = 24 studies, total participants = 17,664) and described interventions delivered at multiple time points up to 90 days following hospital discharge [42]. Interventions such as self-management activities (Relative Risk (RR) 0.81 [0.74, 0.89]), telephone follow-up (RR 0.84 [0.73, 0.97]) and medication reconciliation (RR 0.88 [0.81, 0.96]) were statistically associated with reduced hospital readmissions [42]. The effectiveness of these interventions to enable the identification of unintentional prescribing cascades should be explored in future research.

This research highlighted the importance of greater collaboration between community pharmacists, GPs, patients and secondary care physicians. To achieve this increased collaboration, the different stakeholder groups highlighted the importance of timely information sharing to support safer prescribing. To this end, information and communication infrastructure and national polices that address professional role expansion have the potential to support care delivery. Healthcare professionals and professional representatives interviewed all highlighted the need for a shared electronic health record accessible by healthcare professionals and patients. This would enable timely information sharing across care transitions. In Ireland, there are plans to introduce a national shared care record, but this is not in operation currently [43]. GP-based pharmacists were mentioned by policymakers, professional representatives, GPs and pharmacists in this stakeholder analysis as having a potential role in supporting the management of complex polypharmacy in primary care. In the UK, the role of pharmacists in primary care is established, and in Ireland pilot studies of GP-based pharmacists have been conducted [44–46]. Recent research with GPs in Ireland indicates that there is an interest and appetite for the introduction of clinical pharmacists in General Practice [47, 48].

## Conclusion

This stakeholder analysis highlights several key challenges experienced by patients, carers and clinicians in managing polypharmacy and identifying prescribing cascades. Stakeholders articulated the delicate risk-to-benefit balance in managing multimorbidity with multiple long-term medicines and considered transitions of care following hospital discharge as a time of increased risk for medication-related problems.

## Supplementary Material

aa-23-2312-File002_afae116
