# Supplementary material for: Stakeholder perceptions of and attitudes towards problematic polypharmacy and prescribing cascades: a qualitative study
Source: Age Ageing. 2024 Jun 9;53(6):afae116. doi: 10.1093/ageing/afae116 (PMC11162292; doi:10.1093/ageing/afae116)
Supplement: aa-23-2312-File002_afae116 [file aa-23-2312-file002_afae116.docx]

**Appendix 1 - COREQ (COnsolidated criteria for REporting Qualitative research) Checklist**

A checklist of items that should be included in reports of qualitative research. You must report the page number in your manuscript where you consider each of the items listed in this checklist. If you have not included this information, either revise your manuscript accordingly before submitting or note N/A.

| **Topic** | **Item No.** | **Guide Questions/Description** | **Reported on Page No.** |
| --- | --- | --- | --- |
| **Domain 1: Research team and reflexivity** |  |  |  |
| *Personal characteristics* |  |  |  |
| Interviewer/facilitator | 1 | Which author/s conducted the interview or focus group? | See Data Collection section of Methods |
| Credentials | 2 | What were the researcher’s credentials? E.g. PhD, MD | See Analysis & Reflexivity section of Methods |
| Occupation | 3 | What was their occupation at the time of the study? | See Analysis & Reflexivity section of Methods |
| Gender | 4 | Was the researcher male or female? | Not documented as not relevant to the subject matter. |
| Experience and training | 5 | What experience or training did the researcher have? | See Analysis & Reflexivity section of Methods |
| *Relationship with participants* |  |  |  |
| Relationship established | 6 | Was a relationship established prior to study commencement? | See Analysis & Reflexivity section of Methods |
| Participant knowledge of the interviewer | 7 | What did the participants know about the researcher? e.g. personal goals, reasons for doing the research | See Analysis & Reflexivity section of Methods |
| Interviewer characteristics | 8 | What characteristics were reported about the inter viewer/facilitator? e.g. Bias, assumptions, reasons and interests in the research topic | See Analysis & Reflexivity section of Methods |
| **Domain 2: Study design** |  |  |  |
| *Theoretical framework* |  |  |  |
| Methodological orientation and Theory | 9 | What methodological orientation was stated to underpin the study? e.g.  grounded theory, discourse analysis, ethnography, phenomenology, content analysis | See intro to methods section |
| *Participant selection* |  |  |  |
| Sampling | 10 | How were participants selected? e.g. purposive, convenience, consecutive, snowball | See sampling and recruitment sub -section of the Methods |
| Method of approach | 11 | How were participants approached? e.g. face-to-face, telephone, mail, email | See sampling and recruitment subsection of the Methods |
| Sample size | 12 | How many participants were in the study? | See results section – opening section and Table 1 |
| Non-participation | 13 | How many people refused to participate or dropped out? Reasons? | N/A |
| *Setting* |  |  |  |
| Setting of data collection | 14 | Where was the data collected? e.g. home, clinic, workplace | See data collection sub-section of methods |
| Presence of nonparticipants | 15 | Was anyone else present besides the participants and researchers? | N/A |
| Description of sample | 16 | What are the important characteristics of the sample? e.g. demographic data, date | See Table 1 in Results section |
| *Data collection* |  |  |  |
| Interview guide | 17 | Were questions, prompts, guides provided by the authors? Was it pilot tested? | See Fig 1 |
| Repeat interviews | 18 | Were repeat inter views carried out? If yes, how many? | N/A |
| Audio/visual recording | 19 | Did the research use audio or visual recording to collect the data? | See data collection sub-section of methods |
| Field notes | 20 | Were field notes made during and/or after the inter view or focus group? | See analysis and reflexivity sub-section of methods |
| Duration | 21 | What was the duration of the inter views or focus group? | See results section and Table 1 |
| Data saturation | 22 | Was data saturation discussed? | N/A – study used thematic analysis for data analysis as outlined by Braun & Clarke- data saturation techniques not recommended. |
| Transcripts returned | 23 | Were transcripts returned to participants for comment and/or correction? | See data collection sub-section of methods |
| **Topic** | **Item No.** | **Guide Questions/Description** | **Reported on Page No.** |
|  |  |  |  |
| **Domain 3: analysis and findings** |  |  |  |
| *Data analysis* |  |  |  |
| Number of data coders | 24 | How many data coders coded the data? | See analysis and reflexivity sub-section of methods |
| Description of the coding tree | 25 | Did authors provide a description of the coding tree? | No – thematic analysis employed |
| Derivation of themes | 26 | Were themes identified in advance or derived from the data? | Thematic analysis employed, therefore, codes derived from the data |
| Software | 27 | What software, if applicable, was used to manage the data? | See analysis and reflexivity sub-section of methods |
| Participant checking | 28 | Did participants provide feedback on the findings? | See data collection sub-section of methods |
| *Reporting* |  |  |  |
| Quotations presented | 29 | Were participant quotations presented to illustrate the themes/findings?  Was each quotation identified? e.g. participant number | See Results Section |
| Data and findings consistent | 30 | Was there consistency between the data presented and the findings? | See Results and Discussion Sections |
| Clarity of major themes | 31 | Were major themes clearly presented in the findings? | See Results Section |
| Clarity of minor themes | 32 | Is there a description of diverse cases or discussion of minor themes? | See Results Section |

Developed from: Tong A, Sainsbury P, Craig J. Consolidated criteria for reporting qualitative research (COREQ): a 32-item checklist for interviews and focus groups. *International Journal for Quality in Health Care*. 2007. Volume 19, Number 6: pp. 349 – 357

**Once you have completed this checklist, please save a copy and upload it as part of your submission. DO NOT** **include this checklist as part of the main manuscript document. It must be uploaded as a separate file.**

**Appendix 2**

Interview Topic Guide

**Preamble**

Thank you for agreeing to take part in this study. My name is X and today I hope to hear about your thoughts and experiences about managing medicines for older people. I am going to ask you a series of questions but want to reassure you there are no right or wrong answers. The interview will last no more than one hour but if at any time you would like to take a break or to stop the interview, let me know and we can do that. I’m going to be taking notes whilst recording the interview but that is just to help my memory.

| **Area to be covered** | **Questions** |
| --- | --- |
| Problematic polypharmacy | **Patients/carers**  Let’s start with a little about you…  How old are you?  Can you tell me how many medications you take/the person you care for take at the moment?  *Prompts:* Do you feel this is a lot of medications?  Could you tell me a little about your experience of managing multiple medications?  *Prompts: How do you feel about the number/types of medications you/the person you care for take?*  *Can you describe how/when you take your medicines and what your routine for this?*  *How does having to take you multiple medications impact you? (patient)/How does having to manage multiple medications for someone else impact you? (carer)*  *Do you take all your prescribed medications? If not have you discussed this with anyone?*  Who are the people who prescribe your medicines? Is it just your GP or are there other doctors involved e.g. a consultant?  Who would you contact if you were having problems with your medicines?  **Healthcare professional stakeholder**  Could you tell me a little about your experience of managing patients with multiple medications?  *Prompts: How many medicines does your average older patient take? How do you feel about the number/types of medications you prescribe/dispense?* |
| Risks and benefits | **All participants**  Could you tell me what you believe are the benefits of taking multiple medications?  *Prompts: to you, to other people, to the healthcare system*  **Patients/carers**  Do you know what each of your/their medicines is for? (patient/carer)  **All participants**  Can you tell me what you believe are the challenges of taking multiple medications?  *Prompts: to you, to other people, to the healthcare system*  Can you tell me what you believe are the risks of taking multiple medications?  *Prompts: to you, to other people, to the healthcare system*  What do you think could be done differently to improve the management of multiple medications for older people? |
| Understanding ADRs | Now we are going to move on and discuss adverse drug reactions.  **All participants**  What is your understanding of an adverse drug reaction?  *Prompts: Give definition of adverse drug reaction as “any unintended response to a medicine related to any dose”. Give an example of an adverse drug reaction.*  **Patients/carers**  What is your experience of adverse drug reactions?  *Prompts: Have you or anyone you know experienced an adverse drug reaction?*  *If patient/carer responds that they have experienced an adverse drug reaction:*  Can you tell me a little about what you experienced and how this made you feel?  *Prompts: What was the drug reaction that occurred and how bad was it? Was there anyone that you reported the side effects to? What were your expectations about reporting the reaction? Did you look for any information or support from anyone at that time? Was the medication stopped? How might this experience affect medication you take in the future?*  *If participant indicates little or no experience of adverse drug reactions*  Can you tell me how you might feel about experiencing an adverse drug reaction?  *Prompts: scared, nervous, not bothered…How might having side effects make you feel? Would it impact on taking future medication maybe? What would you do if it happened? Who would you talk to?*  Can you make any suggestions as to how adverse drug reactions could be prevented?  **Healthcare professional stakeholder**  Have you had experience of patients/carers experiencing an adverse drug reaction?  *Prompts: What did you do in response? How did you discuss the reaction with the patient? Did you complete a report to the relevant authority? Why/why not? What was the outcome of reporting? Was there anyone you discussed this experience with or did you seek information or support at that time?*  *If participant indicates little or no experience of adverse drug reactions* Why do you think you haven’t encountered this?  What types of adverse drug reactions do you think might occur for older people who take multiple medications?  *Prompts: How do you think this might impact on an older person?*  Can you make any suggestions as to how adverse drug reactions could be prevented? |
| Prescribing cascade | **All participants**  Now we are going to move on to another topic and discuss prescribing cascades.  A prescribing cascade is when a medication is used to treat the side effect of another medication. *If necessary give an example of a prescribing cascade.*  Sometimes a prescribing cascade is appropriate, for example a PPI may be co-prescribed with a NSAID to reduce the risk of stomach problems. But sometimes it could have been prevented if the first medication was seen as the problem and stopped or adjusted.  **Patients/carers**  Based on this explanation, can you tell me what you think about prescribing cascades?  Can you tell me a little about any experience you may have of a prescribing cascade?  *Prompts: Do you think this has happened for you? What medication was it and what side effect did you experience? What did you do about it? Did you discuss it with anyone? What happened next?*  *If the patient/carer is unsure if they have experienced a prescribing cascade and has provided a medication list the interviewer (a pharmacist) will review the list at this point to screen for potential prescribing cascades and pose relevant questions. For example if on a NSAID and a PPI a question regarding the reason for the PPI could be posed.*  *If no experience of a prescribing cascade:*  In general, what do you do when you think you are having a side effect to a medication?  *Prompts: Would you stop a medication without taking to a healthcare professional like your doctor or pharmacist?*  **Healthcare professional stakeholder**  Do you encounter prescribing cascades a lot in practice?  *Prompts: Can you talk me through the steps involved? How was it identified and managed? Would these be intentional or unintentional cascades that you are referring to?*  **All participants**  What do you think might contribute to prescribing cascades occurring for older people?  What do you think are risks associated with prescribing cascades for older people?  Do you think there are any potential benefits of prescribing cascades for older people?  *Prompt: Sometimes patients are prescribed a medication to prevent side effects of another medication, known as an intentional prescribing cascade. What do you think about this as a practice?*  *If the participant discusses having experience of a prescribing cascade:*  Thinking back, what might have helped to identify that it was a prescribing cascade?  *Prompts: what could you or healthcare professionals have done differently? How could this be prevented in the future?*  *If the participant indicates they have no experience of prescribing cascades:*  Is there anything you think could be done to help identify prescribing cascades among older people?  *Prompts: What things might help e.g. a list of common side effects?*  *Is there anything you think patients, doctors, pharmacists could do?*  In general, what do you think could be done differently to prevent, manage or resolve prescribing cascades?  *Prompts: behaviours, actions etc. Who do you think can help with this?* |
| Concluding questions | In summary, is there anything else you would like to add that hasn’t been discussed so far? |

**Conclusion**

Thank you for your time and for taking part in our research study. We would like to give you the opportunity to review the transcript of your interview today. This review will allow you to amend any errors, provide clarifications and redact sections but won't be able to add anything new to your transcript. Are you happy for us to contact you again when this is available for review?
